# Vertebral osteomyelitis caused by the novel pathogen *Cutibacterium modestum*: a case report

**DOI:** 10.1186/s12879-022-07290-w

**Published:** 2022-03-29

**Authors:** Hirokazu Toyoshima, Kaori Tanaka, Motoaki Tanigawa, Naoto Masuda, Chiaki Ishiguro, Hiroyuki Tanaka, Yuki Nakanishi, Shigetoshi Sakabe

**Affiliations:** 1 Department of Infectious Diseases, Japanese Red Cross Ise Hospital, 1-471-2, Funae, Ise, Mie 516-8512 Japan; 2 Division of Anaerobic Research, Life Science Research Center, Gifu University, 1-1, Yanagido, Gifu, Gifu 501-1194 Japan; 3 Department of Respiratory Medicine, Japanese Red Cross Ise Hospital, 1-471-2, Funae, Ise, Mie 516-8512 Japan; 4 Department of Medical Technology, Japanese Red Cross Ise Hospital, 1-471-2, Funae, Ise, Mie 516-8512 Japan

**Keywords:** *Cutibacterium modestum*, Vertebral osteomyelitis, MALDI, Microbial biochemistry, 16S rRNA gene sequencing, Heterogeneous, Case report

## Abstract

**Background:**

*Cutibacterium modestum* is one of the five species of the genus *Cutibacterium*. While *C. acnes* has been reported as an important pathogen in bone and joint infections, the clinical characteristics of *C. modestum* infections remain unclear*.* Moreover, thus far, there has been no clinical case report regarding *C. modestum* infections.

**Case presentation:**

An 82-year-old man with a history of repeated trigger point injections for lumbago at the L4 level presented with fever and an exacerbation of lumbago. Physical examination indicated knocking pain at the L4–L5 levels; magnetic resonance imaging showed irregular bone destruction of the L4 vertebral body, and low T1 and high T2 intensity lesions at the L4–L5 intervertebral disc. Two sets of blood cultures (two aerobic and two anaerobic) were performed. Intravenous cefazolin was administered, considering the common pathogens of vertebral osteomyelitis, such as *Staphylococcus aureus*. The patient’s condition did not improve; thereafter, anaerobic culture bottles revealed Gram-positive rods on day 11 of incubation. There was no evidence of infective endocarditis upon transthoracic echocardiography. Needle aspiration from the L4–L5 intervertebral disc was performed on day 13 that also showed the presence of Gram-positive rods. The patient was diagnosed with vertebral osteomyelitis caused by *C. modestum* using a combination of characteristic peak analysis with matrix-assisted laser desorption ionization (MALDI), microbial biochemistry examinations, and 16S rRNA gene sequencing from the blood and pus cultures. He was successfully treated with alternative intravenous ampicillin, followed by oral amoxicillin for 10 weeks, according to the tests for ampicillin susceptibility, with a minimum inhibitory concentration of 0.016 μg/mL using E-test® under aerobic conditions.

**Conclusions:**

*Cutibacterium modestum* is a microorganism that is difficult to identify. A combination of characteristic peaks with MALDI, appropriate microbial biochemical examinations, and 16S rRNA gene sequencing may serve as an efficient guide for the identification of *C. modestum*.

## Background

Bacterial species from the genus *Cutibacterium* are Gram-positive, non-spore forming, non-motile rods, which were reclassified from *Propionibacterium* species, based on whole-genome sequencing results [1, 2]. *Cutibacterium modestum*, which was previously known as *Propionibacterium humerusii*, is one of the five species of the genus *Cutibacterium*; it was first reported in 2011 [3]. There is no clinical case report on *C. modestum* infections, and the clinical characteristics of *C. modestum* infections remain unclear. However, *C. acnes*, which belongs to the same subspecies as *C. modestum*, is well recognized as an important pathogen in bone and joint infections, especially in joints with implants [4, 5]. There are methods to identify *C. modestum* microbiologically [6]; however, a comprehensive analysis using various methods is essential for identifying *C. modestum*.

## Case presentation

An 82-year-old Japanese man, with a history of repeated trigger point injections for lumbago at the L4 level for the past 6 months, presented with fever and exacerbation of his lumbago within the previous month.

**Fig. 1:**
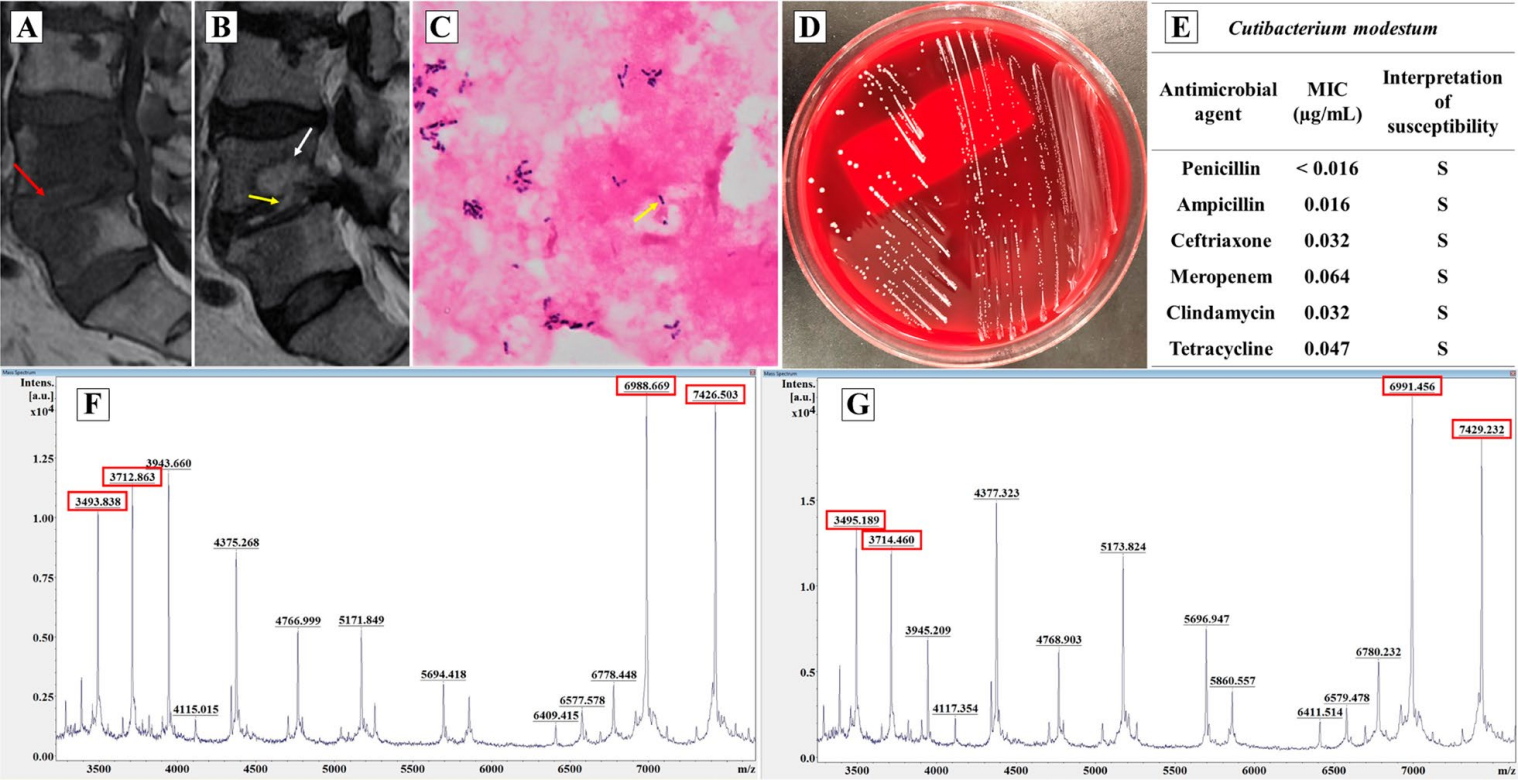
Radiological and microbiological findings. **A**, **B** MRI showed irregular bone destruction at the L4 vertebral body endplate (white arrow) with a T2-weighted image (**B**) and a lesion with low T1 (**A**) and high T2 intensity (**B**) at the L4 vertebral body and L4–L5 intervertebral disc (red and yellow arrows). **C**–**E** Gram staining (×1000) revealed Gram-positive rods (yellow arrow) (**C**). Non-hemolytic and circular white shiny colonies were observed (**D**). The susceptibility testing of the isolates from blood culture using the E-test® (bioMérieux) revealed susceptibility to penicillin and ampicillin, with MICs of less than 0.016 and 0.016 µg/mL, respectively, according to the Clinical and Laboratory Standards Institute criteria (M100-S31) (**E**). **F**, **G** The MALDI spectrum of the isolate obtained from blood (**F**) and pus (**G**) revealed dominant peaks at 3494, 3713, 6989, and 7427 m/z and 3495, 3714, 6991, and 7429 m/z, respectively. These peaks were not seen for other *Cutibacterium* species, including *C. acnes*

Physical examination indicated knocking pain at the L4–L5 levels. The laboratory findings were as follows: C-reactive protein, 20.4 mg/L; and white blood cell count, 7200/µL with 57.1% neutrophils. Magnetic resonance imaging (MRI) showed irregular bone destruction at the L4 vertebral body endplate and a lesion with low T1 and high T2 intensity at the L4 vertebral body and the L4–L5 intervertebral disc (Fig. 1A, B). There was no evidence of infective endocarditis upon transthoracic echocardiography.

Two sets of blood cultures (two aerobic and anaerobic) were performed, and intravenous cefazolin (1 g) was administered every 12 h, considering possible infection with *S. aureus*, a pathogen of vertebral osteomyelitis. He developed pyrexia with persistent lumbago following admission; however, blood culture results remained negative until day 10. Anaerobic culture bottles showed positive results on day 11 of incubation, despite negative results in the aerobic bottles; Gram-positive rods were observed (Fig. 1C). Needle aspiration from the L4–L5 intervertebral disc was performed on day 13, and Gram-positive rods were observed. The isolates were cultured on trypticase soy agar with 5% sheep blood (Nihon Becton–Dickinson, Tokyo, Japan) for 5 days at 37 °C under anaerobic conditions; they formed non-hemolytic and circular white shiny colonies (Fig. 1D). The isolates from blood and pus cultures were identified as *C. modestum* using 16S rRNA gene sequencing, and analysis using the GenBank Basic Local Alignment Search Tool indicated 99.9% (with identities 1433/1435, gaps 0/1435) and 100% (with identities 1417/1417, gaps 0/1417) similarity to *C. modestum*, (GenBank accession no. LC466959), respectively [7]. The isolates showed susceptibility to ampicillin, with a minimum inhibitory concentration of 0.016 µg/mL using E-test® (bioMérieux, Marcy I’Etoile, France) under anaerobic conditions (Fig. 1E).

**Fig. 2:**
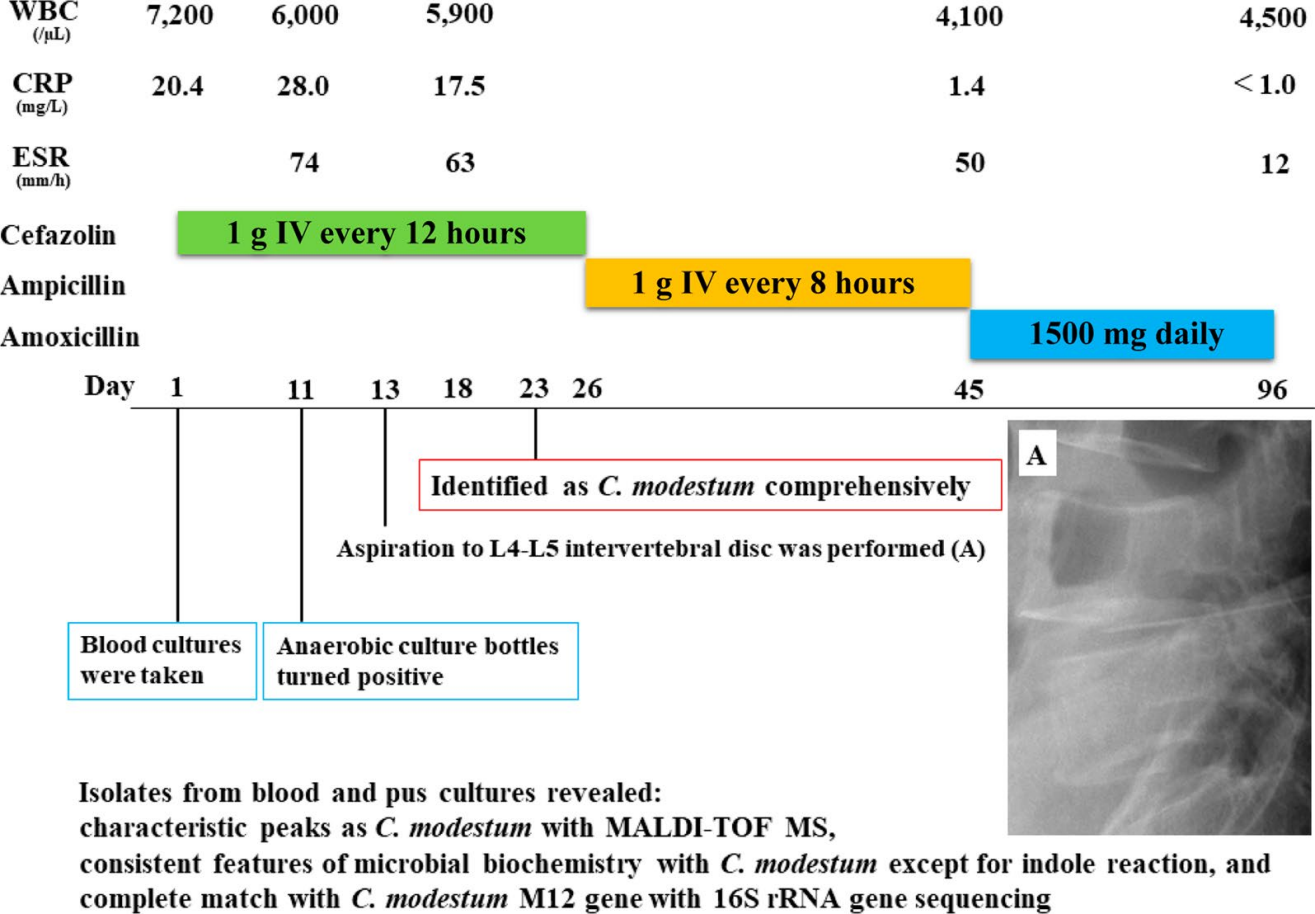
The clinical course of the patient

Our patient was diagnosed with vertebral osteomyelitis caused by *C. modestum*, and cefazolin was replaced with intravenous ampicillin (2 g) administration every 6 h, based on the result of the susceptibility test on day 26 (Fig. 2). He became apyrexial with relief from lumbago and was discharged with a prescription for oral amoxicillin (1500 mg) every day, on day 45. Amoxicillin was discontinued on day 96, and the patient remained disease-free without recurrence and sequelae.

## Discussion and conclusions

There are three clinical issues: (1) the possibility of vertebral osteomyelitis caused by *C. modestum* in clinical settings, (2) methods to identify *C. modestum* and the potential of heterogeneity of *C. modestum*, and (3) clinical management of vertebral osteomyelitis caused by *C. modestum*.

*Cutibacterium acnes* is widely recognized as a post-operatively contracted causative agent of vertebral osteomyelitis [8]. *C. modestum* is an anaerobic, aerotolerant, and non-spore-forming Gram-positive rod [6]. The isolates form non-hemolytic, circular white shiny colonies that are 1.0 × 1.5 mm in size on trypticase soy agar with 5% sheep blood (Nihon Becton–Dickinson) after culture for 5–7 days at 30–37 °C under anaerobic conditions [6]. *C. modestum* is a skin commensal, similar to *C. acnes*; therefore, *C. modestum* could also cause infections via contaminated skin surfaces [1, 6]. Our patient underwent repeated trigger point injections before admission. This is the most plausible source of the infection in this case. *C. modestum* could be misidentified as *C. acnes*, based on the low score value of ≤ 1.70 when using matrix-assisted laser desorption ionization (MALDI) [6]. The database of the MALDI biotyper® (Bruker Daltonik GmbH, Bremen, Germany) lacks the spectrum of *C. modestum* [6]. Additionally, *C. modestum* shows 98.0% similarity to *C. acnes* in 16S rRNA gene sequencing [6]. Therefore, several *C. acnes* infections in previously published case reports could have been *C. modestum* infections.

There are three keys to identify *C. modestum*. First, the dominant peaks in the MALDI spectrum of *C. modestum* are present at 3493, 3712, 6986, and 7424 m/z, which are absent in that of *C. acnes* [6, 9]. This suggests the involvement of *C. modestum* in cases with other *Cutibacterium* species, based on the low scores in MALDI. In our case, the isolates from blood and pus showed a low score value of 1.55 and 1.45, respectively, to *C. acnes* with MALDI biotyper® (Bruker). However, the MALDI spectrum of isolates from blood and pus exhibited dominant peaks of 3494, 3713, 6989, and 7427 m/z and 3495, 3714, 6991, and 7429 m/z, respectively (Fig. 1F, G).

Second, *C. acnes* is divided into three subspecies: *C. acnes* subspecies *acnes*, *C. acnes* subspecies *defendens*, and *C. acnes* subspecies *elongatum*. Unlike *C. modestum*, *C. acnes* subspecies *acnes* and *C. acnes* subspecies *defendens* grow well even under aerobic conditions [6]. *C. modestum* could also grow aerobically; however, the growth is limited, similar to that of *C. acnes* subspecies *elongatum* [6]. This could be used to distinguish *C. modestum* from other *Cutibacterium* species, which show similarity upon 16S rRNA gene sequencing.

Third, the microbial biochemistry of *C. modestum* is unique. *C. modestum* (LC466959) shows negative results for hydrolysis of *N*-acetyl-β-glucosaminidase, indole, phenylalanine arylamidase, leucine arylamidase, pyrazinamidase, β-glucuronidase, β-galactosidase, and gelatin and for ribose fermentation; the other *Cutibacterium* species show variable results [6]. This is useful in identifying *C. modestum*. In our case, the isolates were positive for indole hydrolysis and negative for hydrolysis of other test substrates. This suggests that *C. modestum* could comprise heterogeneous strains.

These characteristics, in addition to the culture period, characteristic colonies, and the finding of Gram staining, contribute to the rapid identification of *C. modestum*, even in cases without 16S rRNA gene sequencing.

Clinically, blood cultures are often negative in *C. acnes* vertebral osteomyelitis [10]. Therefore, blood cultures could yield negative results for *C. modestum* vertebral osteomyelitis. In our case, the anaerobic culture bottles revealed positive results after 11 days of incubation with no preceding antimicrobial administration. Additionally, the needle-aspirated sample from the infected intervertebral disc, obtained on day 13, yielded a positive culture result. The review of 29 *C. acnes* vertebral osteomyelitis cases indicated that the incubation time for the blood cultures was unknown in cases of negative blood cultures [10]. Although this is one case and there is no detailed clinical case report of *C. modestum* vertebral osteomyelitis, these findings suggest the importance of prolonged cultures and local needle aspiration in cases of negative blood cultures for identifying *C. modestum*.

The optimal duration of antimicrobial therapy for *C. modestum* vertebral osteomyelitis is uncertain. In vitro, *C. acnes* could survive on Schaedler medium for 8 months under anaerobic conditions; the conditions are similar in the vertebrae or intervertebral discs [11]. Our patient was successfully treated with the appropriate antibiotics for 10 weeks. Antibiotic therapy for a mean duration of 8.7 weeks (range 2–28 weeks) has resulted in good outcomes in 45/46 (98%) cases, among the 51 reported cases of *C. acnes* vertebral osteomyelitis [10]. This suggests that the duration of antimicrobial therapy in our case was appropriate, despite the results in vitro.

In conclusion, *C. modestum* is a difficult-to-identify pathogen mimicking *C. acnes* with regard to its microbiological characteristics. However, a combination of characteristic peak analysis with MALDI, appropriate microbial biochemistry examinations, and 16S rRNA gene sequencing may serve as a reliable and efficient guide for identifying and diagnosing *C. modestum* infections.

## Data Availability

The datasets generated and/or analysed during the current study are included in the manuscript.

## Abbreviations

MALDI: Matrix-assisted laser desorption ionization
MRI: Magnetic resonance imaging

## References

[1] Scholz CFP, Kilian M. The natural history of cutaneous propionibacteria, and reclassification of selected species within the genus *Propionibacterium* to the proposed novel genera *Acidipropionibacterium* gen. nov., *Cutibacterium* gen. nov. and *Pseudopropionibacterium* gen. nov. Int J Syst Evol Microbiol. 2016;66:4422–32. 10.1099/ijsem.0.001367. 10.1099/ijsem.0.001367 27488827

[2] Nouioui I, Carro L, García-López M, Meier-Kolthoff JP, Woyke T, Kyrpides NC (2018). Genome-based taxonomic classification of the phylum Actinobacteria. Front Microbiol.

[3] Butler-Wu SM, SenGupta DJ, Kittichotirat W, Matsen FA, Bumgarner RE (2011). Genome sequence of a novel species, *Propionibacterium humerusii*. J Bacteriol.

[4] Torrens C, Bellosillo B, Gibert J, Alier A, Santana F, Prim N (2022). Are *Cutibacterium acnes* present at the end of primary shoulder prosthetic surgeries responsible for infection? Prospective study. Eur J Clin Microbiol Infect Dis.

[5] Bumgarner RE, Harrison D, Hsu JE (2020). *Cutibacterium acnes* isolates from deep tissue specimens retrieved during revision shoulder arthroplasty: similar colony morphology does not indicate clonality. J Clin Microbiol.

[6] Dekio I, Sakamoto M, Suzuki T, Yuki M, Kinoshita S, Murakami Y, et al. *Cutibacterium modestum* sp. nov., isolated from meibum of human meibomian glands, and emended descriptions of *Cutibacterium granulosum* and *Cutibacterium namnetense*. Int J Syst Evol Microbiol. 2020;70:2457–62. 10.1099/ijsem.0.004058. 10.1099/ijsem.0.004058 32559834

[7] Suzuki MT, Giovannoni SJ (1996). Bias caused by template annealing in the amplification of mixtures of 16S rRNA genes by PCR. Appl Environ Microbiol.

[8] Desoutter S, Cottier JP, Ghout I, Issartel B, Dinh A, Martin A (2015). Susceptibility pattern of microorganisms isolated by percutaneous needle biopsy in nonbacteremic pyogenic vertebral osteomyelitis. Antimicrob Agents Chemother.

[9] Dekio I, McDowell A, Sakamoto M, Tomida S, Ohkuma M. Proposal of new combination, *Cutibacterium acnes* subsp. *elongatum* comb. nov., and emended descriptions of the genus *Cutibacterium*, *Cutibacterium acnes* subsp. *acnes* and *Cutibacterium acnes* subsp. *defendens*. Int J Syst Evol Microbiol. 2019;69:1087–92. 10.1099/ijsem.0.003274. 10.1099/ijsem.0.003274 30762517

[10] Uçkay I, Dinh A, Vauthey L, Asseray N, Passuti N, Rottman M (2010). Spondylodiscitis due to *Propionibacterium acnes*: report of twenty-nine cases and a review of the literature. Clin Microbiol Infect.

[11] Csukás Z, Banizs B, Rozgonyi F (2004). Studies on the cytotoxic effects of *Propionibacterium acnes* strains isolated from cornea. Microb Pathog.

